# Enhanced cortical responsiveness during natural sleep in freely behaving mice

**DOI:** 10.1038/s41598-020-59151-8

**Published:** 2020-02-10

**Authors:** Sumire Matsumoto, Kaoru Ohyama, Javier Díaz, Masashi Yanagisawa, Robert W. Greene, Kaspar E. Vogt

**Affiliations:** 1 International Institute for Integrative Sleep Medicine (WPI-IIIS), University of Tsukuba, Tsukuba, Japan; 2 School of Integrative and Global Majors, University of Tsukuba, Tsukuba, Japan; 3 Japan Society for the Promotion of Science Research Fellow, Tokyo, Japan; 4 Department of Psychiatry & Neuroscience, Peter O’Donnell Brain Institute, UT Southwestern Medical Center, Dallas, TX USA

**Keywords:** Sleep, Slow-wave sleep

## Abstract

Cortical networks exhibit large shifts in spontaneous dynamics depending on the vigilance state. Waking and rapid eye movement (REM) sleep are characterized by ongoing irregular activity of cortical neurons while during slow wave sleep (SWS) these neurons show synchronous alterations between silent (OFF) and active (ON) periods. The network dynamics underlying these phenomena are not fully understood. Additional information about the state of cortical networks can be obtained by evaluating evoked cortical responses during the sleep-wake cycle. We measured local field potentials (LFP) and multi-unit activity (MUA) in the cortex in response to repeated brief optogenetic stimulation of thalamocortical afferents. Both LFP and MUA responses were considerably increased in sleep compared to waking, with larger responses during SWS than during REM sleep. The strongly increased cortical response in SWS is discussed within the context of SWS-associated neuro-modulatory tone that may reduce feedforward inhibition. Responses to stimuli were larger during SWS-OFF periods than during SWS-ON periods. SWS responses showed clear daily fluctuation correlated to light-dark cycle, but no reaction to increased sleep need following sleep deprivation. Potential homeostatic synaptic plasticity was either absent or masked by large vigilance-state effects.

## Introduction

Behaviorally, sleep is characterized by loss of consciousness, increased arousal threshold, and immobility^1^. Despite decreased communication with the periphery during sleep, cortical neurons remain almost as active as during waking, although the pattern is strikingly different. The activity switches from ongoing irregular action potential firing during waking to synchronized rhythmic oscillations between silent (OFF) and active (ON) periods^2,3^, called slow wave activity in non-rapid eye movement (NREM) sleep or slow wave sleep (SWS), indicating a profound change in the functional cortical architecture. The underlying mechanisms of this switch and the consequences for cortical function and behavior are not fully understood.

In addition to observations of spontaneous oscillations, measurements of evoked cortical responses can be used to probe the functional state of the cortical network during waking and sleep^4–11^. Sensory stimuli can reach the cortex during SWS, which allows for studying the effect of wake-sleep transitions on cortical responsiveness^7–10^. Cortical reactivity indeed changes between waking and sleep, but the effect is strongly dependent on the sensory modality and type of stimulus. In the somatosensory cortex, both response depression and enhancement are observed during sleep compared with waking. In human subjects, sensory evoked potentials are smaller during NREM sleep compared with waking^7^. In macaques, responses to tactile stimuli are significantly decreased during SWS compared with waking^8^. Electrical activation of the medial lemniscus results in smaller responses in SWS compared with those in the preceding waking episode^11^. On the other hand, whisker deflections in rats produce larger evoked responses, but exhibit faster adaptation to repeated stimuli during SWS compared with waking^9^. Auditory stimulation in rats evokes cortical responses that are comparable across vigilance states^10^. Sensory stimuli or pre-thalamic stimulation involves subcortical structures and does not solely reflect changes in cortical circuits across sleep-wake states. The complexity of the pathway may cause those inconsistencies.

Direct activation of cortex or thalamocortical afferents makes it possible to circumvent any effect occurring at pre-thalamic pathways. Electrical stimulation of the visual thalamus in cats shows decreased cortical responses during SWS compared with waking and REM sleep^12^. In human subjects, transcranial magnetic stimulation (TMS) was performed combined with high-density EEG recording^6,13,14^. They observed larger evoked cortical response in sleep compared with wake, while the propagation of the evoked activity beyond the stimulation site was smaller. No definitive explanation for the increased responses was provided so far^14^, due to the limit of the resolution of evoked signals and the neuronal activity underlying this phenomenon is not clear.

We investigated the reaction of the cortical network to repeated, brief optogenetic thalamocortical activations to understand the precise neuronal activity during the response across vigilance states. Cortical local field potentials (LFP) and multi-unit activity (MUA) from primary motor cortex (M1) were measured to capture not only the direct thalamocortical effects, but also the spread of excitation within the cortex in freely behaving mice over prolonged periods of time to ensure consistent delivery of stimuli across many natural sleep-wake transitions. We found that cortical responses were profoundly increased between waking and SWS, but the increase was less pronounced between waking and REM sleep. Transitions were rapid and involved both cortical LFP and MUA. Within-SWS responses did not change greatly between ON and OFF periods of slow wave activity. Over a 24-h period, we observed a fluctuation correlated to light-dark cycle in SWS responses, but no purely sleep need dependent modulation.

## Results

**Figure 1:**
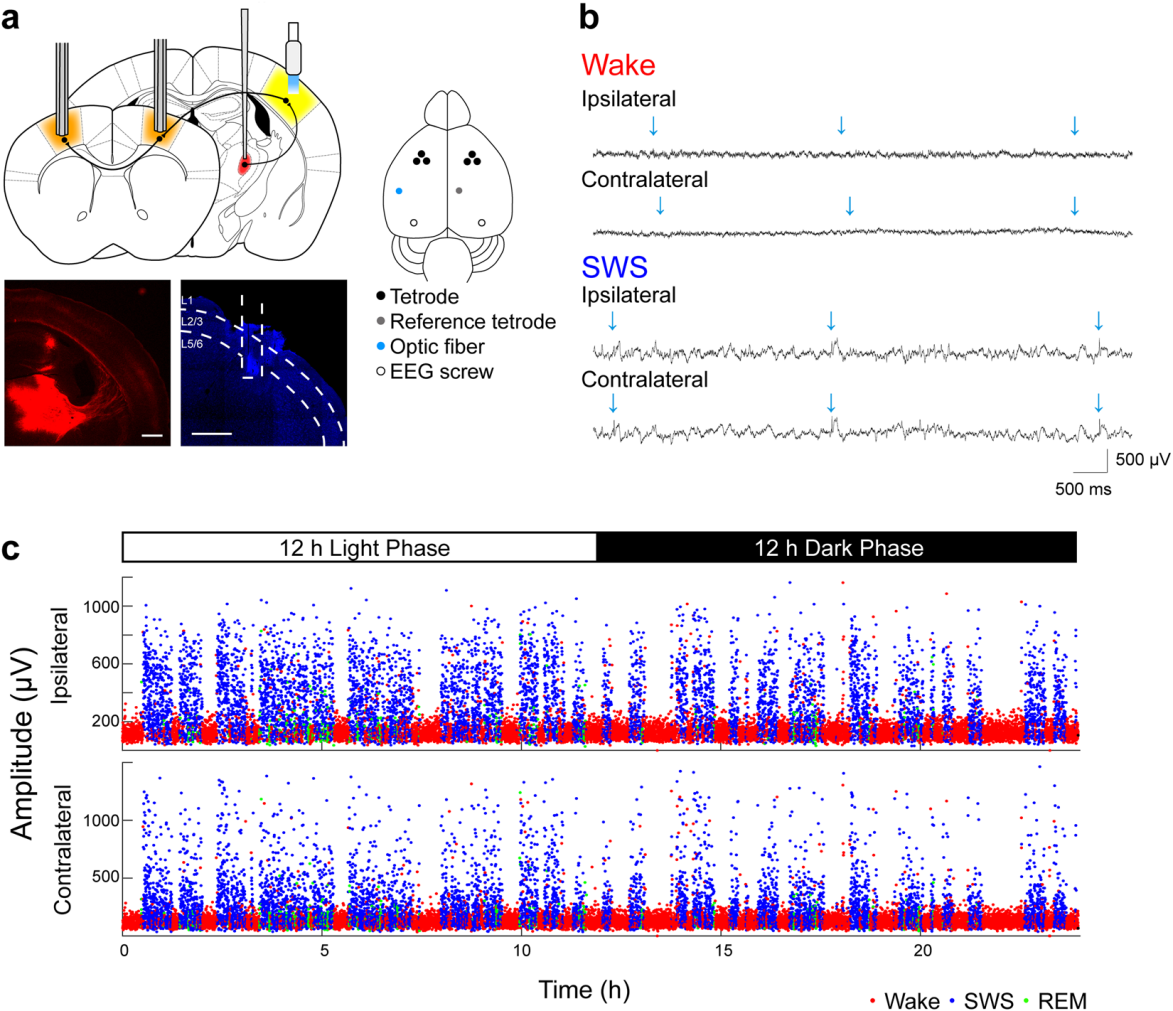
Chronic Optogenetic Probing of Cortical Responses. (**a**) Schematic of virus injection, and placement of optogenetic stimulation and tetrode recordings. AAV-ChR2-mCherry was injected into the ventral posteromedial nucleus of the thalamus, an optic fiber was implanted in S1, and three tetrodes were implanted in M1 of each hemisphere. Coronal sections of forebrain stained against mCherry (red) showing the thalamic injection and cortical innervation, and a detailed coronal cortical section (blue) showing the electrode track. Scale bars: 500 μm. (**b**) Examples of local field potential (LFP) signals with 5-ms optical stimulation every 5 ± 1 s during wake and SWS. Green arrows: stimulation time-point. (**c**) Representative plot of LFP response amplitude over 24 h from both an ipsilateral and contralateral tetrode in response to unilateral optogenetic stimulation (approximately 17,280 stimuli). Response amplitudes were calculated as the 10^th^ to 90^th^ percentile difference in LFP signals in a 30-ms time window following stimulation. Plots are color coded: red = wake, blue = SWS, and green = REM sleep.

Thalamic input to the cortex provides a well-characterized pathway for evoking controlled cortical responses^15–17^. We injected adeno-associated virus containing a plasmid to express channelrhodpsin-2 (ChR2) into the somatosensory thalamus (Fig. 1a). At the same time, animals were implanted with a multi-tetrode drive system over the ipsi- and contralateral M1 and a light-guide was implanted over S1 (Fig. 1a). In rodents, S1 has both anatomic^15^ and functional^18–22^ connections with M1. This recording system therefore allowed us to study synapticaly connected networks involving cortical connection from S1 to M1 and M1 to contralateral M1 connections - a mainly monosynaptic excitatory commissure through the corpus callosum. After recovery from surgery and expression of ChR2 that could evoke clear and stable responses (Fig. 1a), mice were habituated to the recording chamber and the recording setup. Optogenetic stimuli (5 ms) were delivered at pseudo-random intervals of 5 ± 1 s (Fig. 1b). These stimuli produced time-locked responses in the LFP recorded by our tetrodes (Fig. 1b). Total time spent in wake, in SWS and REM sleep (Supplementary Fig. S1), or in their epoch durations did not differ significantly between stimulation and non-stimulation conditions (Mean ± standard deviation(SD) of time in control versus stimulus conditions [paired t-test], wake: 12.4 ± 0.9 h versus 10.3 ± 1.7  h [N = 4, *p* = 0.11], SWS: 10.5 ± 1.1 h versus 12.5 ± 1.4 h [N = 4, *p* = 0.10], REM: 1.1 ± 1.7 h versus 1.2 ± 0.4 h [N = 4, *p* = 0.07]). Thus, optogenetic stimulation did not significantly affect the wake sleep pattern.

**Figure 2:**
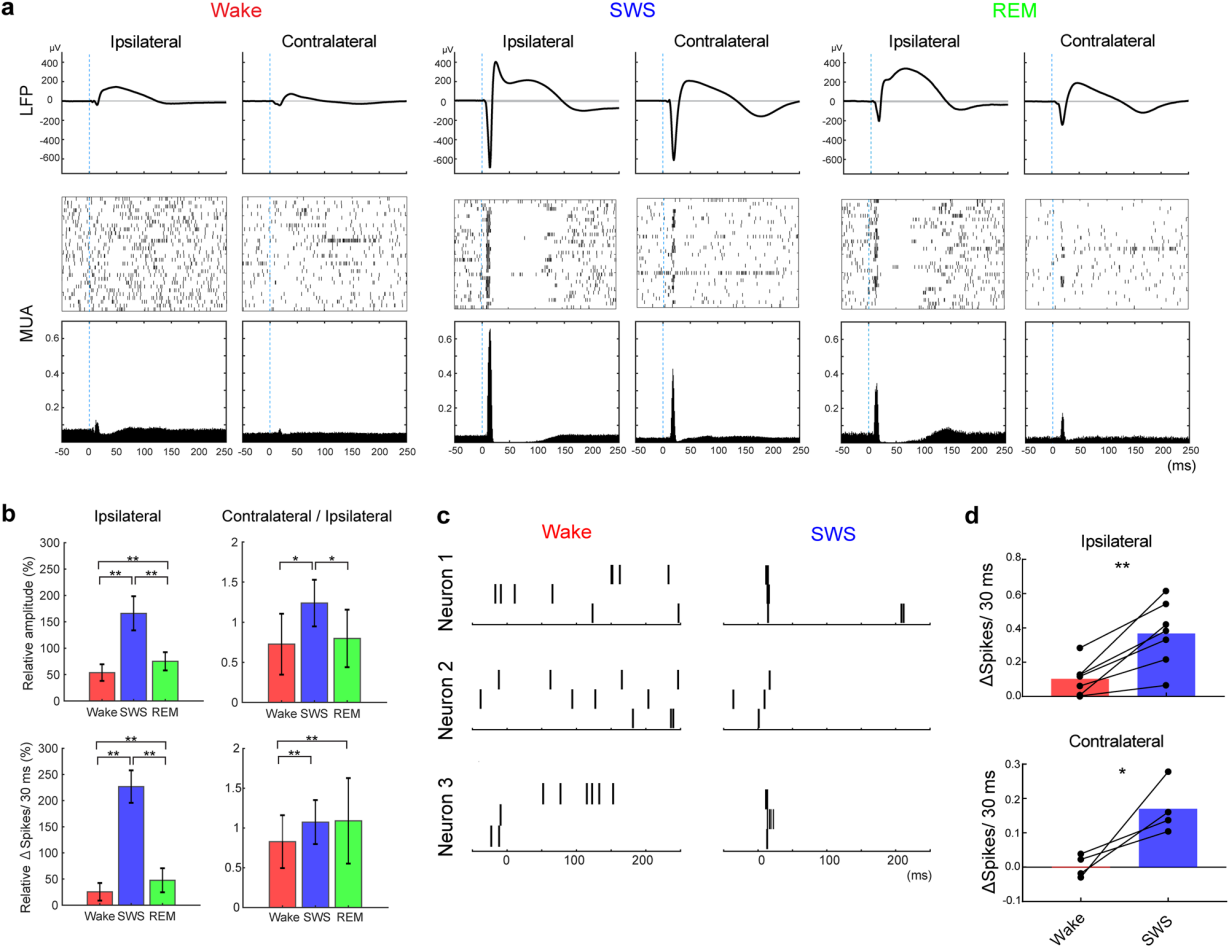
Cortical Responses across Vigilance State. (**a**) Example of averaged time-locked LFP signals and MUA in each vigilance state. For LFPs, signals from −50 ms before to 250 ms after stimulation were extracted and averaged (gray line: mean ± 3 SD of pre-stimuli LFP signals, top). For MUA, the time-points of unit activity were extracted in the same time windows as for the LFP. Examples of 30 stimulations are shown as a raster plot (middle). Peri-stimulus time histograms (1-ms bin size) were calculated from the MUA data (bottom). Both LFP- and MUA-responses were larger during sleep compared with wake. (**b**) Summary of response sizes for both LFP relative to the mean across states (left top) and MUA relative to the mean across states (left bottom, 4 animals, 12 tetrodes from ipsilateral to the activated thalamic input, 12 tetrodes from contralateral). Intracortical response (right two panel) was calculated as the average LFP and MUA response sizes of the three contralateral tetrodes divided by the average of the ipsilateral tetrodes for each stimulus (error bar: SD, *p < 0.05, **p < 0.01 [12 tetrodes, paired t-test]). MUA were calculated as the number of spikes within 30 ms after stimulation minus baseline activity. (**c**) Example raster plot of three single units following three stimulations (Neuron 1 and 2 are from ipsilateral, Neuron 3 is from contralateral tetrode). In SWS, single neurons reliably responded to the stimulation. (**d**) Changes of single units across wake and SWS. Baseline-corrected number of unit activity within 30 ms after stimulation is shown (*p < 0.05, **p < 0.01 [Ipsilateral: 7 units, contralateral: 4 units, paired t-test]).

Vigilance state transitions produced rapid changes in the LFP response amplitude (Fig. 1c, Supplementary Fig. S2), with notably large responses during SWS (Fig. 1b,c). On average, SWS LFP responses were several-fold larger compared with waking (Fig. 2a,b), while REM responses were still significantly larger compared with waking, but much smaller than SWS responses (Fig. 2a,b) on both the ipsi- and contralateral sides of the cortical stimulation. These differences in the LFP response amplitude were also reflected in stimulus-evoked MUA (Fig. 2a). The correlation coefficient between the LFP response amplitude and the MUA response was 0.34 ± 0.04 in wake and 0.75 ± 0.22 in SWS (p < 0.01 [48 tetrodes, Pearson’s correlation coefficient]).

MUA responses were biphasic in all vigilance states. A brief (30 ms) window with increased unit activity after the optogenetic stimulus was followed by a longer period of reduced unit activity.We defined inhibition as the deficit (number of action potentials missing) below baseline in the 400 ms window following the excitatory phase (30–430 ms post stimulus) and excitation as the number of action potentials above baseline in the 30 ms window immediately following the stimulus. The ratio (inhibition divided by excitation) was 55.5 ± 30.1% for waking, 61.1 ± 39.4% for SWS, and 55.7 ± 30.7% for REM sleep on the ipsilateral and 40.1 ± 21.7% for waking, 49.1 ± 45.7% for SWS, and 68.3 ± 62.8% for REM sleep on the contralateral side. The ratio did not differ significantly between wake and SWS on both the ipsilateral (*p* = 0.33) and contralateral side (*p* = 0.12 [12 tetrodes each, paired t-test]).

In a subset of experiments, we isolated single units from the MUA response from two mice (Fig. 2c,d). On the basis of their waveform, the neurons were classified as 6 putative excitatory and one putative inhibitory neurons on the ipsilateral side and 3 excitatory and one inhibitory neurons on the contralateral side (see Material and Methods). In all cases, the single units also exhibited increased evoked activity in SWS compared with waking (Fig. 2d, *p < 0.05, **p < 0.01 [paired t-test]).

**Figure 3:**
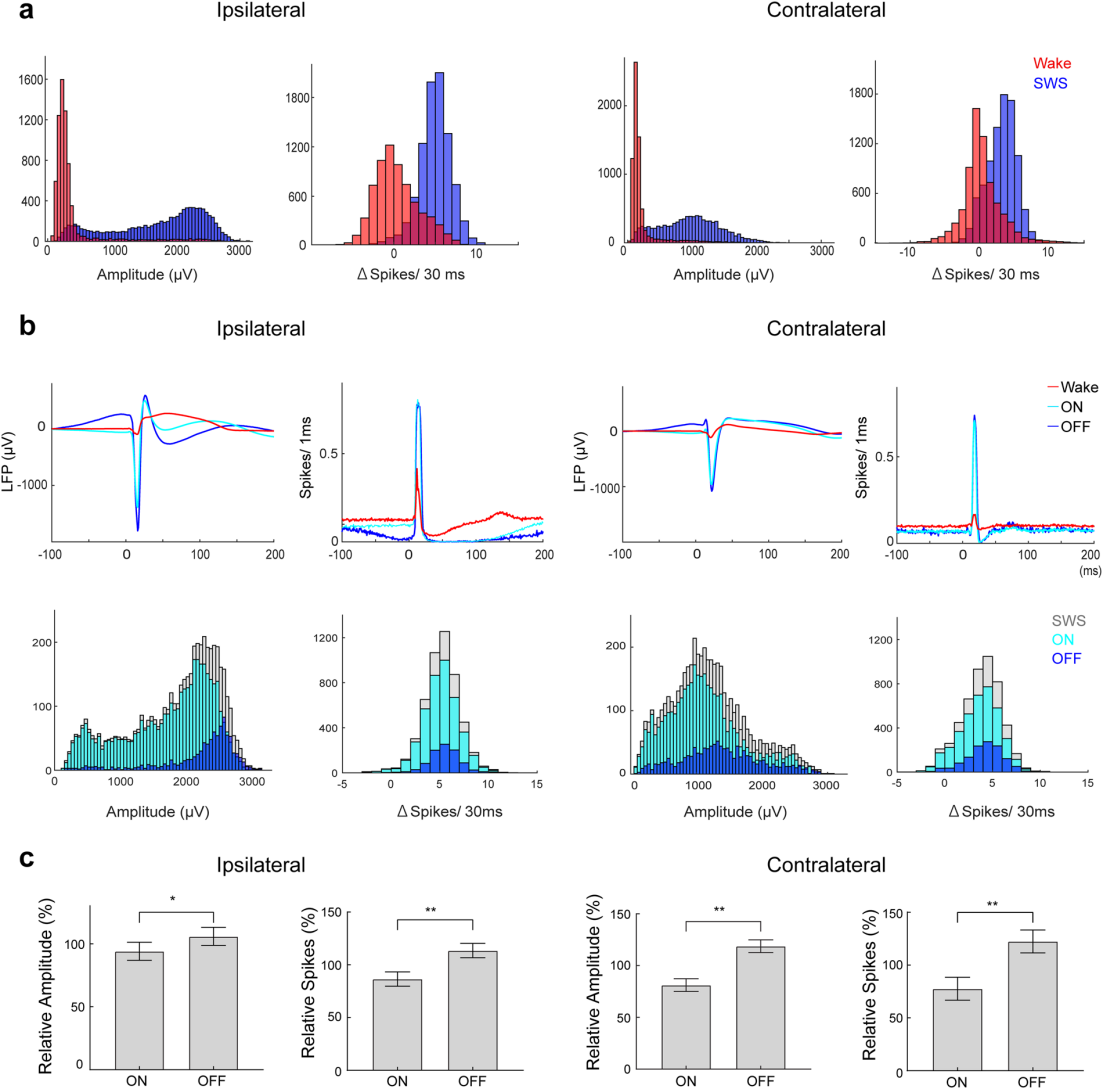
Response Dynamics Across ON/OFF Phases of SWS Slow Oscillation. (**a**) Histogram of the distribution of LFP response and evoked unit activity in wake and SWS (bin width: 50 μV for LFP, 1 spike for units). (**b**) Traces of mean LFP and MUA responses during wake, SWS-ON, and SWS-OFF phase (red: wake, light blue: ON, dark blue: OFF, gray: all SWS). Stimuli were delivered every 5 ± 1 s and post-hoc separated into ON- and OFF- phase (see Methods) depending on LFP and MUA immediately preceding the stimulus for each tetrode. (**c**) Summary of the difference of the response during ON/OFF phase (mean of the medians in each tetrode for LFP amplitude and mean for MUA were normalized to the arithmetic mean of ON and OFF response, error bar: SD, *p < 0.05, **p < 0.01 [N = 4, paired t-test]).

We also observed a wide distribution of LFP response amplitudes between waking and SWS (Fig. 3a). These fluctuations in the cortical response during SWS might be linked to ON/OFF oscillations, which are hypothesized to be caused by shifts in excitability^23,24^, and thus the response amplitude. We therefore separated the evoked responses into ON and OFF according to the LFP preceding the optogenetic stimulation (see Materials and Methods; Fig. 3b). Indeed, spiking activity just before the stimulus onset was significantly lower for OFF responses compared with ON responses, confirming the validity of the LFP-based classification (Fig. 3b). Surprisingly, evoked LFP responses were slightly, but significantly, larger for OFF responses compared with ON responses (Fig. 3c), and the evoked unit activity was also higher for OFF responses compared with ON responses (Fig. 3c). The weak differences between ON/OFF evoked LFP responses did not fully explain the higher responsiveness or wide distribution because the difference in the oscillation did not fully separate large and small responses and both were larger than that during waking. This finding also suggests that OFF periods in the cortex do not prevent the activation of synapses or make it more difficult to evoke action potentials in cortical neurons.

**Figure 4:**
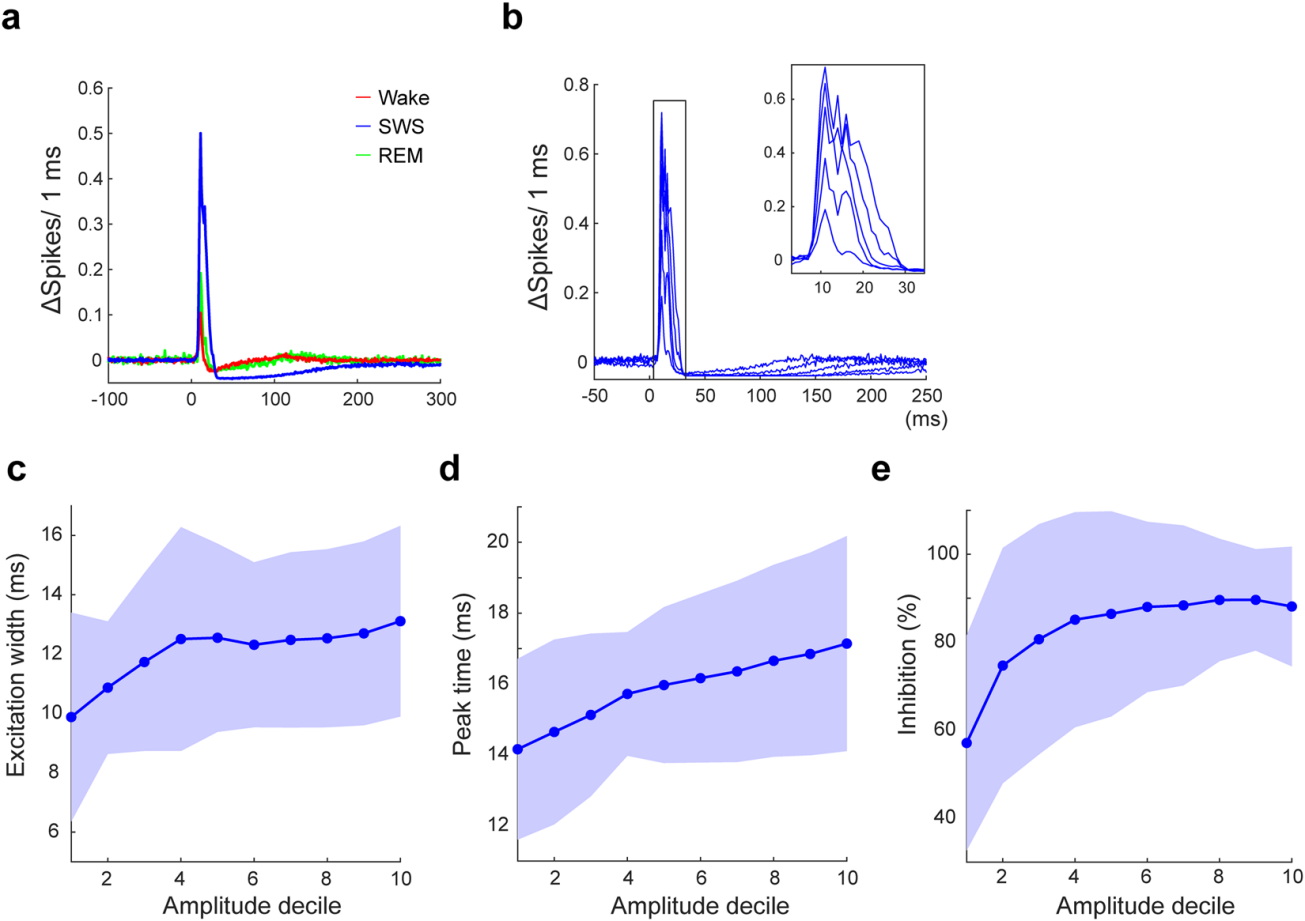
Reduced Feedforward Inhibition in Larger Responses. (**a**) Peri-stimulus time histograms (1 ms bin size) of MUA in each state. (**b**) Histograms of quintile of SWS MUA response. (**c**–**e**) Development of the action potential firing time-course as a function of the response amplitude (in deciles). (**c**) Excitation width of the response, defined as full-width at half-maximum of the excitation peak in the histogram (Mean ± SD). (**d**) The response peak time, defined as the bin with the largest number of action potentials. (**e**) The percentage of inhibition defined as the % of the reduction of number of unit activity from 50 to 100 ms after stimulation to the number of unit activity of the time window from 150 to 50 ms before the stimulation.

Evaluation of the MUA responses allows for more precise interpretation of the behavior of cortical neurons. A larger MUA response was accompanied by a wider excitation peak, both across vigilance states (Fig. 4a) and within SWS (Fig. 4b). In SWS, the decile of the response on the basis of the evoked LFP amplitude indicated that a small response occurred in a shorter time window and a larger response occurred in a longer time window (Fig. 4c) with a longer time to peak (Fig. 4d). In addition, the large excitatory transients were followed by a longer period of inhibition (Fig. 4e).

This observation indicates that the overall excitation-inhibition relationship is stable for both small and large responses. The duration of the spiking window in the cortex, however, is largely determined by feedforward inhibition^25^ - the wider spiking window of larger SWS responses suggests that feedforward inhibition plays an important role in modulating the evoked cortical responses we observed.

**Figure 5:**
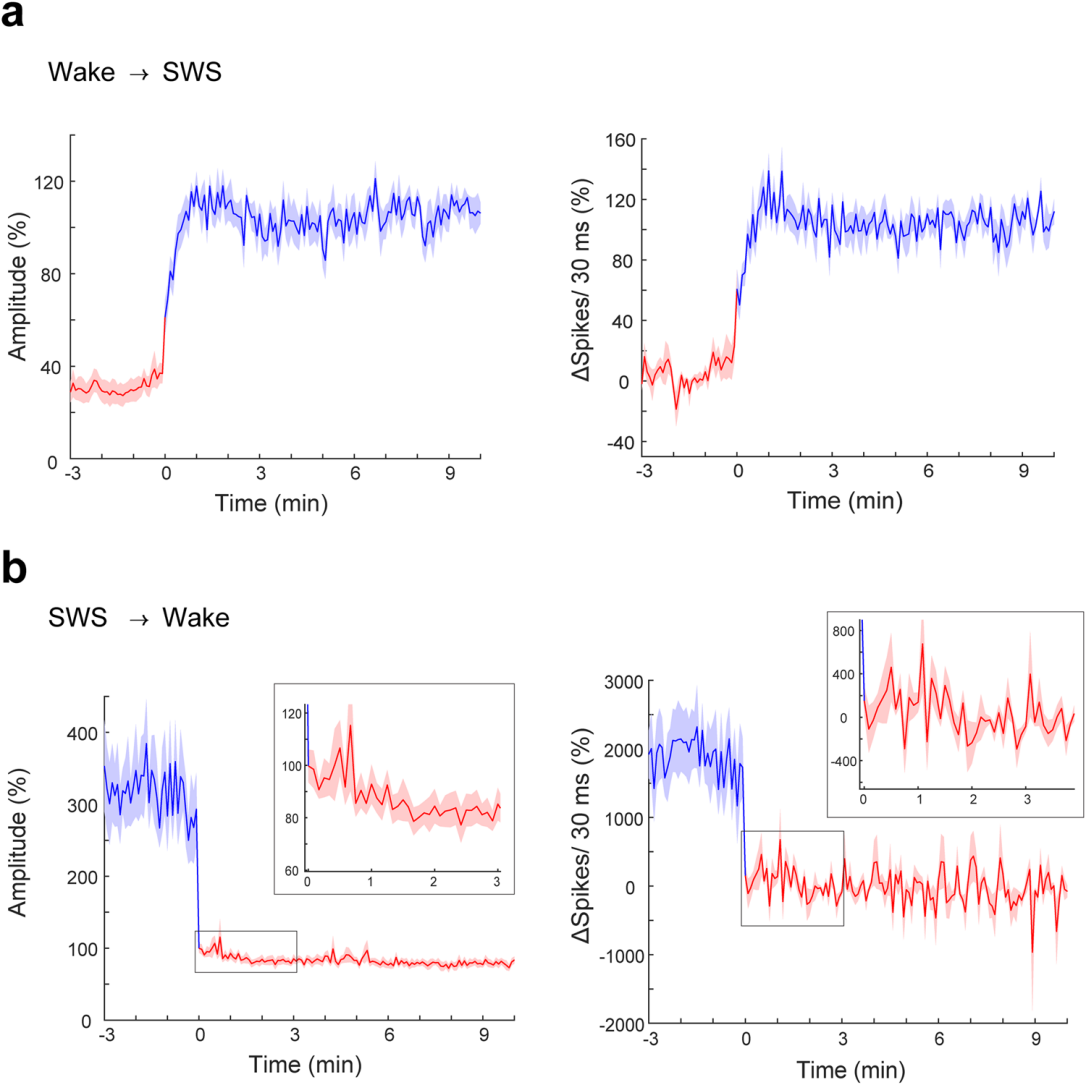
Fast Transition Kinetics of LFP- and MUA- Response Size at Vigilance State Change. Plot of mean LFP and MUA response as a function of time since beginning of the episode. SWS or wake episodes longer than 10 min that occurred after longer than 3 min wake or SWS episodes were selected. LFP and MUA responses were aligned at their transition and LFP-response amplitudes and the MUA response were averaged after normalizing to the whole 13-min mean of each recording (115 wake to SWS and 76 SWS to wake transition from 4 animals, mean ± standard error of the mean (sem)). (**a**) Changes in the cortical response following state transitions took less than a minute in either direction, with the transition from SWS to wake taking the least amount of time. Within SWS cortical responses were highest 30–50 s after detection of the wake-SWS transition. After this peak, the responses slowly decayed by 10–20% over 3 min. (**b**) SWS to waking transitions were faster and showed little adaptation of the response during the subsequent waking episode by 10–20% over 1–2 min.

To reduce the number of potential physiological processes that might underlie the vigilance state-dependent fluctuations, we measured the time-course of waking to SWS and SWS to waking transitions. In general, waking to SWS transitions took several minutes to be fully expressed and stabilized thereafter (Fig. 5a). Small changes in the response amplitude were observed preceding the behavioral transition, but the largest changes in the response amplitude coincided with the behavioral state transition. Responses grew over 2–3 min afterwards, slightly settled over the next 3 min, and then remained stable for the duration of the SWS episode. SWS to waking transitions were faster (Fig. 5b) and showed little adaptation of the response during the subsequent waking episode.

**Figure 6:**
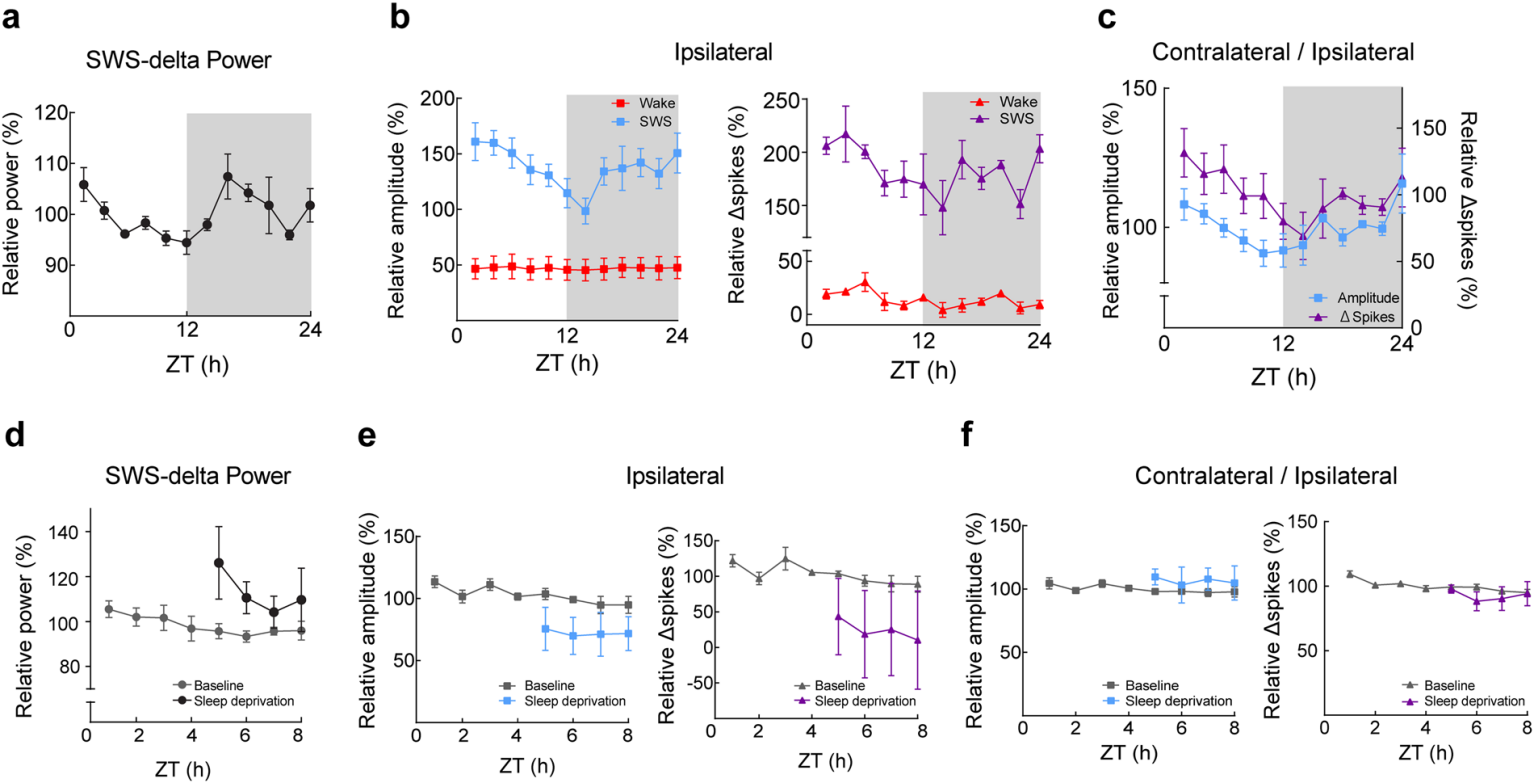
Daily fluctuation on LFP and MUA Response in SWS but not in wake. (**a**) Dynamics of delta power over 24 h recording. (**b**) LFP (left) and MUA (right) responses in SWS (blue) and wake (red) over 24 h. Medians of the measurements over 2 h are expressed as percentage of the 24 h mean (error bar: sem). Note the dynamics correlated to light-dark cycle; decreasing during the light phase and increasing during dark phase for both LFP response amplitude and unit response (LFP amplitude: *p* < 0.05, Spikes: *p* < 0.05, [8 recordings, re*p*eated measurement ANOVA]). (**c**) Contralateral response size divided by the ipsilateral response size over 24 h as intracortical response. (**d**) Delta power was averaged over 1 h windows and plotted against Zeitgeber time (ZT) during recovery sleep after 4 h sleep deprivation start at ZT = 0 (*p* < 0.01, [6 recordings, paired t-test in first hour of recovery sleep]). Gray traces show baseline behavior. (**e**) LFP (left) and MUA (right) response size for 4 h during recovery sleep is plotted against ZT (mean of the medians over 1 h bin in each tetrode for LFP amplitude and mean over 1 h bin for MUA were normalized relative to the mean of baseline, error bar: sem, LFP amplitude: *p* = 0.14, Spikes: *p* = 0.30, [6 recordings, paired t-test in first hour of recovery sleep]). (**f**) LFP (left) and MUA (right) of intracortical response after for 4 h during recovery sleep relative to the mean of baseline plotted against ZT (error bar: sem, LFP amplitude: *p* = 0.24, Spikes: *p* = 0.45 [3 recordings, paired t-test in first hour of recovery sleep]).

Considerable evidence indicates a vigilance-state dependent modulation of synaptic strength^5,26–28^; the synaptic homeostasis hypothesis postulates that synaptic strength during waking generally and gradually increases with sleep need and is then homeostatically downregulated in subsequent SWS episodes^29,30^. SWS delta power is the most reliable indicator of sleep need^31^. We observed a well-known oscillation in delta power^31^ in the LFP recordings, driven by homeostatic sleep need, (Fig. 6a) that peaked at the beginning of the light phase, which is the resting phase for nocturnal animals such as mice. The LFP and MUA responses showed a similar oscillation correlated to light-dark cycle in SWS, while no such oscillation was observed for the waking responses. The median response amplitude every 2 h in each state is shown in Fig. 6b. The number of spikes evoked by optogenetic stimulation also showed this daily fluctuation (Fig. 6b).

This modulation was also visible in the ratio between the ipsi- and contralateral responses, where the ratio was largest at the beginning of the light phase for both evoked LFP responses and the number of evoked MUA responses (Fig. 6c). Thus, parallel to the daily fluctuation in sleep need, we observed an oscillation in cortical evoked LFP responses and evoked MUA.

To distinguish between a coincident circadian effect and a true sleep need dependence, we artificially increased the sleep need by inducing a 4-h sleep deprivation beginning at the start of the light phase. Compared with undisturbed control recording, sleep deprivation produced a significant increase in SWS delta power in the subsequent recovery sleep (Fig. 6d), indicating that sleep deprivation indeed increased the sleep need. Optogenetically evoked LFP response amplitudes did not show a sleep need dependent increase over unperturbed control recordings (Fig. 6e). Similarly, the MUA response did not differ significantly between these two conditions (Fig. 6e). Finally, the ratio between the ipsi- and contralateral responses was not affected by the sleep deprivation-induced increase in the sleep need (Fig. 6f). We also evaluated the relationship between the response size and delta power of spontaneous LFP signals just before the stimulation in SWS and found only a weak correlation (correlation coefficient ± SD = Ipsilateral LFP: 0.12 ± 0.06, MUA: 0.10 ± 0.06, Contralateral LFP: 0.13 ± 0.06, MUA: 0.12 ± 0.03; *p* < 0.01 [21 tetrodes in each site, Pearson’s correlation coefficient], see Material and Methods).

## Discussion

We found that cortical LFP and MUA responses were several-fold larger during SWS than during waking, while responses during REM sleep were approximately double the size during waking with vigilance state transitions accompanied by rapid changes in the response amplitude. Larger SWS responses were correlated with longer excitation-time windows, but were only slightly affected by ON and OFF periods of slow oscillations. On a longer time-scale, SWS responses exhibited clear daily fluctuation, peaking at the beginning of the rest phase coincident with highest sleep need. The cortical response during recovery sleep after sleep deprivation, however, was not significantly changed.

The increased cortical responses in SWS compared with waking were consistent with findings from TMS in humans^13,14^. In contrast, sensory evoked responses in humans and animals in natural sleep and under anesthesia typically show smaller and more variable modulation of cortical responses across waking and SWS^7^. For some sensory modalities, no change in cortical responsiveness or even smaller responses in SWS are reported^10^. Sensory evoked responses are typically reduced in REM sleep compared with waking^8^. Recordings of smaller sensory evoked responses in SWS at the thalamic and cortical level support the hypothesis that thalamic filtering significantly contributes to reduced cortical responses^32^. Sensory evoked potentials also show complex adaptive properties; in rats, the response to whisker stimulation in SWS is large for low frequency stimuli and begins to adapt at more than 5 Hz stimuli in SWS^9^. In our stimulation procedure, adaptation was not observed because the stimulus, a pulse every 5 ± 1 s, was too infrequent for adaptation.

Due to the rapid time-course of the observed changes, we hypothesized that the well-described changes in the neuro-modulatory environment between vigilance states significantly contribute to changes in the cortical response amplitude. Waking is characterized by high monoaminergic and cholinergic tone, SWS by low monoaminergic and cholinergic tone, and REM sleep by high cholinergic, but low monoaminergic tone^33^. Acetylcholine (ACh) inhibits principal neurons in layers II to V in the cortex^34^ particularly in spiny stellate neurons and their recurrent network^35,36^. This might be a major contributor to the increased responses in SWS compared with waking. In addition, activation of cholinergic receptors in the piriform cortex inhibits intrinsic connections, but not extrinsic long-range connections^37^. In summary, high cholinergic activity in the cortex suppresses cortical connectivity through both muscarinic and nicotinic receptor activation. Noradrenaline effects on cortical neurons are also inhibitory *in vitro*^38^ and *in vivo*^39^, and noradrenaline predominantly inhibits sensory cortex neurons. Interestingly, ACh excites thalamic neurons^40^ and increases the response reliability of thalamocortical projection neurons^41^. The combination of both these effects shapes the cortical response to sensory-evoked cortical responses and may explain the discrepancy between sensory evoked stimuli, which involve thalamic circuitry, and direct cortical stimulation, which does not.

Cortical MUA showed clear biphasic responses to optogenetic stimuli. A rapid increase in activity was followed by a more prolonged reduction, which then returned to baseline with or without a rebound. The reduced activity may be caused by a number of factors, such as pre- and postsynaptic inhibition or synaptic fatigue - our data does not allow a differentiation. For the sake of simplicity we refer to this phenomenon as inhibition. The ratio between the number of unit activity added in the excitatory phase and those missing during inhibition did not change significantly between vigilance states, paralleling prior studies on spontaneous excitation/inhibition balance in waking and SWS^42^.

Thalamocortical afferents to somatosensory cortex reliably evoke feedforward inhibition^43^ and a reduction in feedforward inhibition leads to a significant broadening of the time window for cortical action potential (AP) generation, illustrating the role of feedforward inhibition in shaping the permissive window for AP generation in cortical circuits^25^. In our results, larger responses in SWS showed a significantly wider time window for spiking. Although it is not possible to definitively differentiate between the contribution of feedforward and feedback inhibition in shaping the inhibitory response on the basis of our experiments, the results are well explained by a decrease in feedforward inhibition contributing to the generation of large cortical responses. Indeed activation of basal forebrain cholinergic input in cortical slices leads to pronounced disynaptic inhibition^44^ by activating non-fast spiking interneurons via nicotinic ACh receptors^34^. A reduction in cortical ACh in SWS is expected to reduce this inhibitory component. The exact circuit cannot be deduced from our data, however, because multiple interneuron types are sensitive to cholinergic modulation.

Thalamocortical synapses show use-dependent depression *in-vitro*^45^ and *in-vivo*^46^. Thus, decreased spontaneous thalamocortical activity in SWS might contribute to the observed increased cortical response. Thalamocortical circuits in SWS, however, exhibit intermittent rhythmic burst firing with peak activity above the persistent activity in waking, and this activity tends to coincide with cortical UP states^47^. The sizes of ON or OFF responses differed only very moderately. In addition, thalamocortical projection neurons are as active in REM sleep as they are in waking - leaving the significant increase in cortical responses in REM sleep unexplained. Thus, purely use-dependent modulation of thalamocortical afferents is an unlikely explanation for the changes we observed.

The responses in SWS were not only significantly larger compared to waking, they also exhibited large trial-to-trial variability. We could rule out ON/OFF fluctuations as a major contributor to this phenomenon, but we currently do not know the mechanisms behind the high variability. The mechanisms underlying ON/OFF transitions in SWS are still not well understood. Fluctuations in excitability due to intrinsic mechanisms such as intracellular calcium influx triggering Ca^2+^-dependent K^+^ channels or fluctuations in potassium leak channel availability are postulated^23,24^ to underlie the transition to the DOWN state. Alternatively, or in addition, synaptic exhaustion might produce a functional disconnect between cortical neurons and a transition to the DOWN state^48,49^. The evoked responses were larger in the OFF period compared with the ON period for both the LFP, which mostly reflects synaptic currents, and evoked MUA, which depends on both synaptic input and postsynaptic excitability. Thus, neither the synaptic input to the recorded network nor its reaction to the input in the form of spikes was significantly reduced in the OFF period, indicating that neurons are continuously inhibited and synapses are continuously depressed during the OFF periods. We were unable to determine the precise phase of the OFF period at which stimuli were delivered. It is therefore possible that the mechanisms mentioned above are responsible for triggering transitions, but not for maintaining OFF periods, in accordance with some models of ON/OFF transitions^49^.

The small increase in cortical responses in the OFF period can be explained by two mechanisms. First, the amplitude of postsynaptic potentials of cortico-cortical synapses scale with the driving force and are otherwise independent of the ON/OFF period fluctuation^32^. The increased driving force in the OFF state can thus explain the increase in the LFP amplitude. Second, the increased evoked MUA activity may be due to the almost twofold increase in the input resistance in the OFF period compared with the ON period^2,50^.

TMS in humans, time-locked to the phase of slow oscillation, produces larger responses in the UP state compared with the DOWN state^14,51^, whereas sensory evoked potentials show the opposite; thus, the type of input activated seems to influence its impact during ON/OFF oscillations. Early work on the synaptic responsiveness and excitability of cortical neurons^32^ showed a decreased synaptic response and propensity to spike. The excitatory response generated by the optogenetic stimulus was short-lived and followed by a significantly longer period of inhibition during SWS; thus, the thalamic input may have re-set the slow oscillation^50^. Interestingly, optogenetic stimulation never produced a stable ON period, indicating that ON-period triggering from optogenetic thalamic input is unlikely.

Another possible mechanism of the response size difference is sleep need-dependent control. Within-state analysis of evoked cortical responses revealed a fluctuation within light-dark cycle of the LFP and MUA response size in SWS that paralleled the well-documented oscillation in sleep need. The same fluctuation could not be resolved in waking and we lacked sufficient REM data to analyze it in this manner. According to the synaptic homeostasis hypothesis, cortical synapses are globally strengthened during waking and homeostatically downregulated during SWS^27,28^, and the direction of the fluctuation is compatible with the synaptic homeostasis hypothesis. We therefore also analyzed the response amplitude during wake under sleep deprivation, and found no significant increase (Supplementary Fig. S3). Other mechanisms, however, such as subtle shifts in the neuro modulatory tone, might also play a role because the correlation between the delta power of preceding spontaneous signals and response size was weak. Indeed, sleep deprivation resulted in a clear increase in recovery sleep delta power, indicating increased sleep need; nevertheless, we did not observe a concomitant increase in the cortical response amplitude. If anything, the response was smaller compared to the baseline recording conditions. There are several possible reasons for this surprising result. The simplest explanation is that response amplitude is modulated by a circadian, but not homeostatic mechanism that happens to follow daily sleep need fluctuations. Alternatively, recovery sleep is characterized by strong slow wave activity; although we did not find a depressant effect of the OFF state on cortical response size, an interaction between strong endogenous slow waves and cortical excitability cannot be ruled out. Indeed, previous work has found a period of refractoriness for electrically evoked cortical responses following strong slow waves^52^. Moreover, sleep need increase after sleep deprivation may engage regulatory mechanisms that are not activated during baseline sleep need fluctuations. It is almost impossible to separate the effects of sleep loss from the effects of sleep deprivation-induced stress. Loss of monoaminergic tone in REM sleep resulted in increased responses in our hands; thus, conversely stress-related increases in monoaminergic tone might suppress responses somewhat. Also, the increase in excitatory synaptic transmission observed in SWS is balanced by an upregulation of inhibitory activity. So far, investigations of the synaptic homeostasis hypothesis have mainly focused on excitatory synapses formed on spines and therefore onto principal neurons. The potential involvement of inhibitory interneurons might have been overlooked.

Complex circuit mechanisms ensure the arrival of sensory information at the cortex during SWS. The increase in cortical responsiveness in SWS might constitute a safety mechanism in that signals that make it past the thalamic filter generate strong and reliable responses. Increased cortical network responses may also be necessary for maintaining cortical activity in the absence of sufficient peripheral input.

## Materials and Methods

### Animals

Experimental procedures were approved and carried out in accordance with local and national regulations following approval by the animal care and use committee of the University of Tsukuba. C57BL/6J male mice aged at the range of 13–26 weeks were used for surgery and recorded for about 6 months (mean ± SD age at virus injection: 14.4 ± 1.5 weeks; at implantation of tetrode: 22.4 ± 3.6 weeks; at first recording: 25.8 ± 6.1 weeks, Jackson Laboratory). Mice were single-housed after surgery under a 12-h light/12-h dark cycle with food and water available ad libitum.

### Adeno-associated virus injection

Mice were anesthetized by an intraperitoneal injection of avertin (0.3 ml/kg) and inhalation of isoflurane (4% for induction and 1–2% for maintenance). pAAV10-eF1a-ChR2-mCherry was injected unilaterally into the ventral posteromedial nucleus of the thalamus (−1.86 A/P, +1.8 M/L, −3.5 D/V, 140 nl) in the left hemisphere to produce the expression of ChR2.

### Microdrive implantation

After sufficient recovery from the virus injection (at least 2 weeks), tetrodes were implanted using an electrode manipulator, a so-called microdrive, in addition to an electroencephalography (EEG) skull screw electrode and electromyography (EMG) wire electrodes. The mice were anesthetized with avertin and isoflurane as described above, and the skull was exposed through a skin incision. The surface of the brain was exposed by craniotomy. After removing the dura, a microdrive system with 6 tetrodes (KANTHAL Precision Technology, nichrome, 14 μm diameter) was surgically implanted in mice together with two EEG wires (A-M Systems, silver) attached to stainless steel screws, two EMG electrodes in the neck muscle (Cooner wire), and a ground wire (A-M Systems, stainless steel) attached to stainless steel screw and optical fiber. Three tetrodes each were placed in M1 cortex layer 5 of the right and left hemispheres (1.4 A/P, ±2.0 M/L, −1.4 D/V) and one tetrode was placed into the ventral hippocampal commissure (vhc; −0.9 A/P, 0.8 M/L, −2.4 D/V). An optical fiber was implanted into the primary somatosensory cortex barrel field (S1BF) in the left hemisphere (−4.37 A/P, −0.7 M/L, −1.74 D/V, 30°, THORLABS).

### Extracellular recording

After sufficient recovery from microdrive implantation (at least 2 weeks), the mice were habituated to the recording chamber. The chamber has a suspending recording tether with a pulley system to allow mice to move freely without disturbing their behavior by interference from the tethers, amplifiers, and optical fibers. Data acquisition and online spike detection were performed using 32-ch Digital Lynx 4SX system (Neuralynx). LFP, EEG, and EMG signals were digitized at 1 kHz, after band-pass filtering at 0.1 Hz–10 kHz for LFP, 10–500 Hz for EEG, and 10–1000 Hz for EMG. LFP were recorded from one electrode of each tetrode with the vhc tetrode as a reference. Units were simultaneously recorded from all four leads of the tetrodes and digitized at 32 kHz after band-pass filtering at 600 Hz–6 kHz.

### Optical stimulation

Optical stimulation was performed using a laser controlled by Master-8 (AMPI) or custom-built TTL generator based on Arduino (Arduino). We used an average of 85.6 mW with about 20% of reduction from the laser to the fiber tip attached to the brain. For the single pulse stimulation, a pulse (duration: 5 ms) was automatically delivered every 5 ± 1 s. The total number delivered during the 24 h recording used in the analysis were mean ± SD in all state: 17264.6 ± 28.9, wake: 7319.9 ± 864.2, SWS: 9053.4 ± 727.9, REM: 891.3 ± 477.0.

### Sleep deprivation

Recordings were started at Zeitgeber time (ZT) = 0 (usually at 9 AM) and mice were sleep-deprived for 4 h from ZT = 0 to ZT = 4 by cage change and gentle handling while optogenetic stimulation was ongoing. After sleep deprivation, mice were freely allowed to enter recovery sleep. As baseline we used recordings from the mice in unperturbed condition one day before and in two animals also one day after the sleep deprivation day. Average response amplitude did not change between the pre- and post-sleep deprivation control.

### Histology

The recording site and ChR2 expression were histologically confirmed after completion of all experiments. Mice were anesthetized with avertin (0.3 ml/kg) or chloroform (10%). First, the tissue near the tip of each tetrode was lesioned by direct current injection (30 μA, 7 s into each tetrode) using a current generator to visualize the recording sites after brain slice preparation. After current injection, the mice were transcardially perfused with phosphate-buffered saline (approximately 50 ml/mice) and 10% formalin neutral buffer solution (approximately 50 ml/mouse). The microdrives were removed and the brain tissue carefully removed and immersed in 10% formalin neutral buffer solution at 4 °C overnight for fixation. The next day, the brains were placed into 30% sucrose solution for cryoprotection and embedded into O.C.T. compound (Tissue-Tek) at −80 °C for at least 1 h. Brains were sliced by cryostat at 30 ~ 50 µm and mounted on microscopy slides. ChR2 expression was assessed by observing the mCherry signal without any further staining. Nissl staining was performed overnight at 4 °C to reveal the lesioned recording site (NeuroTrace, 1:500). After washing with phosphate-buffered saline, slices were mounted on slides. ZEISS Axio Zoom. V16 (Carl Zeiss Co., Ltd.) and LSM700 (Carl Zeiss Co., Ltd.) microscopes were used for image acquisition.

### Data analysis

Data were analyzed using custom-written programs in Matlab (Math Works) and Graph Pad Prism 7 (GraphPad Software). Results were considered significant for *p*-values < 0.05.

#### Sleep scoring

Sleep scores were determined using surface EEG signals alone or in combination with LFP and surface EEG signals in combination with EMG signals with Matlab-based sleep scoring software. Each 4-s epoch was staged into Wake, SWS (NREM sleep), and REM sleep. Wake was scored based on low amplitude, fast EEG activity, and high EMG power. SWS was characterized by high delta oscillation and low EMG power. REM sleep was characterized by a theta band-dominated EEG and atonia in the EMG signal.

#### Spike sorting

Single spike sorting was achieved with Spike Sort 3D software (Neuralynx) offline using tetrode data for a maximum of 1 h. Clusters were considered to represent single units if the isolation distance was >=20, L-ratio <=0.3. Units with spike wave forms with a peak-to-peak width less than 250 μs were classified as inhibitory neurons, while units with a longer width were classified as excitatory neurons.

#### Evoked response analysis

The amplitude of an optogenetically evoked LFP was determined from 30 ms time window after each stimulus onset. The 10^th^ to 90^th^ percentile difference in the distribution of samples in this time window of each stimulation was used as the evoked LFP amplitude (Supplementary Fig. S2). An evoked-MUA response was determined by the number of action potentials within 30 ms of the each stimulus presentation after subtracting the basal activity. Basal activity was defined as the sum of APs in a 300-ms time window before stimulus delivery divided by 10, which is the mean spontaneous activity during 30 ms. For vigilance states the values were normalized to the average in each state over 24 h. For ON/OFF state analysis the values were normalized to the mean of all values across ON and OFF states. For sleep deprivation experiments, all values were normalized to the mean of the control condition values.

#### ON/OFF period detection

OFF periods just prior to stimulus delivery were detected offline for each tetrode separately, on the basis of LFP characteristics. Large positive deflections of the LFP 10 ms before stimulation compared to the mean of the preceding 3 s were considered OFF state, if the deflection exceeded a threshold based on prior LFP activity. The threshold was set at three times the SD of the gamma power (20–100 Hz) in the three seconds prior to stimulation. This procedure can detect OFF state onsets more rapidly than methods based on delta band filtered data. All other cases were considered ON period stimulations. When multi-unit activity for such classified ON and OFF responses was analyzed, we found a clear reduction in activity just prior to stimulus delivery (Number of units in 10 ms time widow before stimulation per trial (mean ± SD): ON = 0.4 ± 0.3, OFF = 0.2 ± 0.2, N = 43 (tetrodes), *p* < 0.01, paired t-test; Supplementary Fig. S4).

#### MUA kinetics

Peri-stimulus time histograms in 1-ms bin size calculated from MUA were used for evoked-MUA kinetics analysis. The bin counts of the histogram were divided by the number of traces of each vigilance state and the basal activity (mean of the counts from 200 to 50 ms before the stimulation) was subtracted. Quintiles and Deciles of the response were determined on the basis of the evoked LFP amplitude. The width of the excitation peak was defined as full width of half maximum of the peak in the histogram. The mode of the MUA distribution evaluated between 10–30 ms after stimulation was considered as peak time of the response. The percentage of inhibition defined as the % of the reduction of unit activity at 50–100 ms after stimulation to the unit activity respect to the same time window before the stimulation.

#### State transition detection

State transition data after more than 3 min wake or SWS were collected and post transition state (SWS or wake) durations longer than 10 min were evaluated. LFP and MUA responses from ipsilateral tetrodes were aligned at their transition points and averaged. The responses were normalized on the basis of the mean response size over 13 min in each animal.

#### Analysis of response over 24 h and sleep deprivation

The delta power in SWS was determined as the power in the 0.5–4 Hz range for the 4 s preceding each stimulation time-point. LFP- and MUA- response sizes were first stratified by vigilance states, and binned into 1 or 2 h intervals. The median (LFP amplitude) or the mean (MUA) of response size in each bin were calculated for each tetrode.

## Supplementary information

Supplementary Figures and Legends.

## Data Availability

The datasets generated during the current study are available from the corresponding author on reasonable request.
