# Supplementary material for: Enhanced cortical responsiveness during natural sleep in freely behaving mice
Source: Sci Rep. 2020 Feb 10;10:2278. doi: 10.1038/s41598-020-59151-8 (PMC7010820; doi:10.1038/s41598-020-59151-8)
Supplement: Supplementary file 1 — Supplementary Figures and Legends. [file 41598_2020_59151_MOESM1_ESM.pdf]

Supplementary Information

Enhanced cortical responsiveness during natural sleep in freely behaving mice

Authors

Sumire Matsumoto[1,2], Kaoru Ohyama[1,3], Javier Díaz[1], Masashi Yanagisawa[1], Robert W Greene[1,4], Kaspar E Vogt[1*]

1. International Institute for Integrative Sleep Medicine, University of Tsukuba, Tsukuba, Japan
2. School of Integrative and Global Majors, University of Tsukuba, Tsukuba, Japan
3. Japan Society for the Promotion of Science Research Fellow
4. Department of Psychiatry & Neuroscience, Peter O'Donnell Brain Institute, UT Southwestern Medical Center, Dallas, TX, USA

* Corresponding author (vogt.kaspar.fu@u.tsukuba.ac.jp)

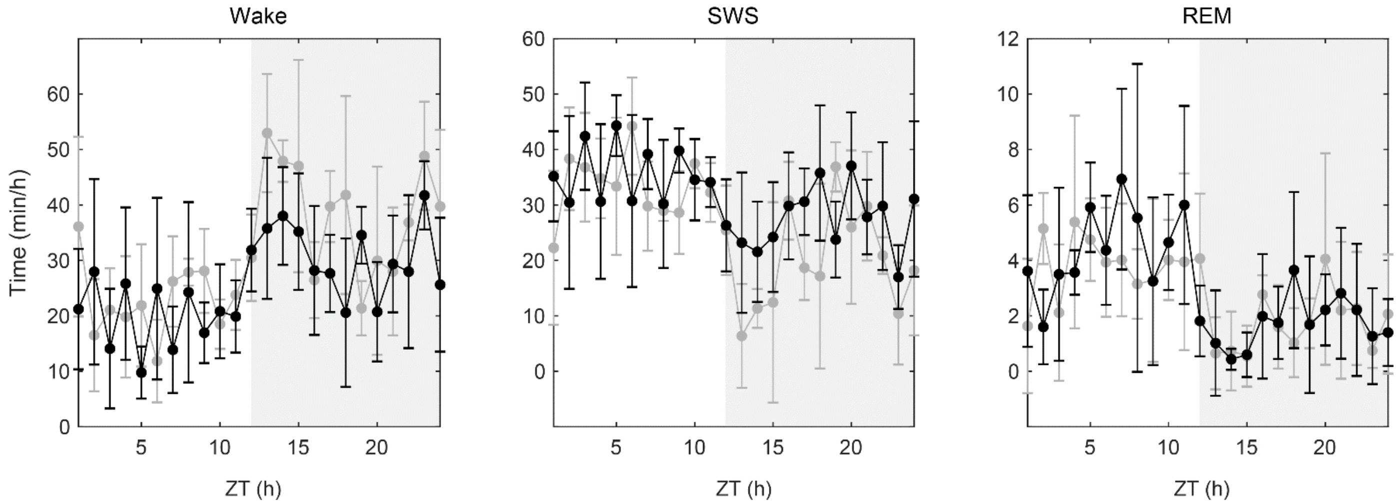

Figure S1.

The average time course of vigilance state across 24 h for animals included in the analysis (gray: no stimulation, black: stimulation, N = 4 for both conditions, error bar: SD, n.s. between no stimulation and stimulation, 2-way repeated measurement anova). Each bin shows the minutes of each state in 1 hour.

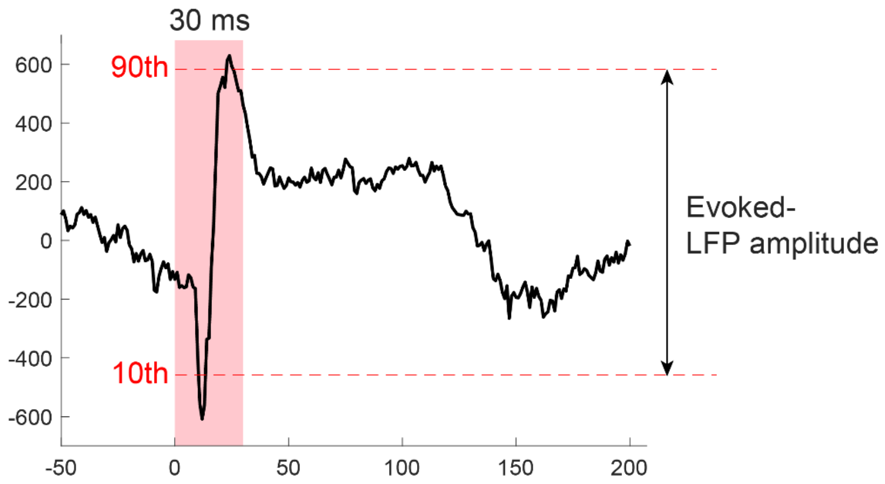

Figure S2.

A representative example of LFP during the light stimulation and the illustration of the measurement process of evoked LFP amplitude. The evoked LFP amplitude was determined from 30 ms time window (red area) after each stimulus onset. The 10th to 90th percentile difference in the distribution of samples (red dash lines) in this time window of each stimulation was used as the evoked LFP amplitude.

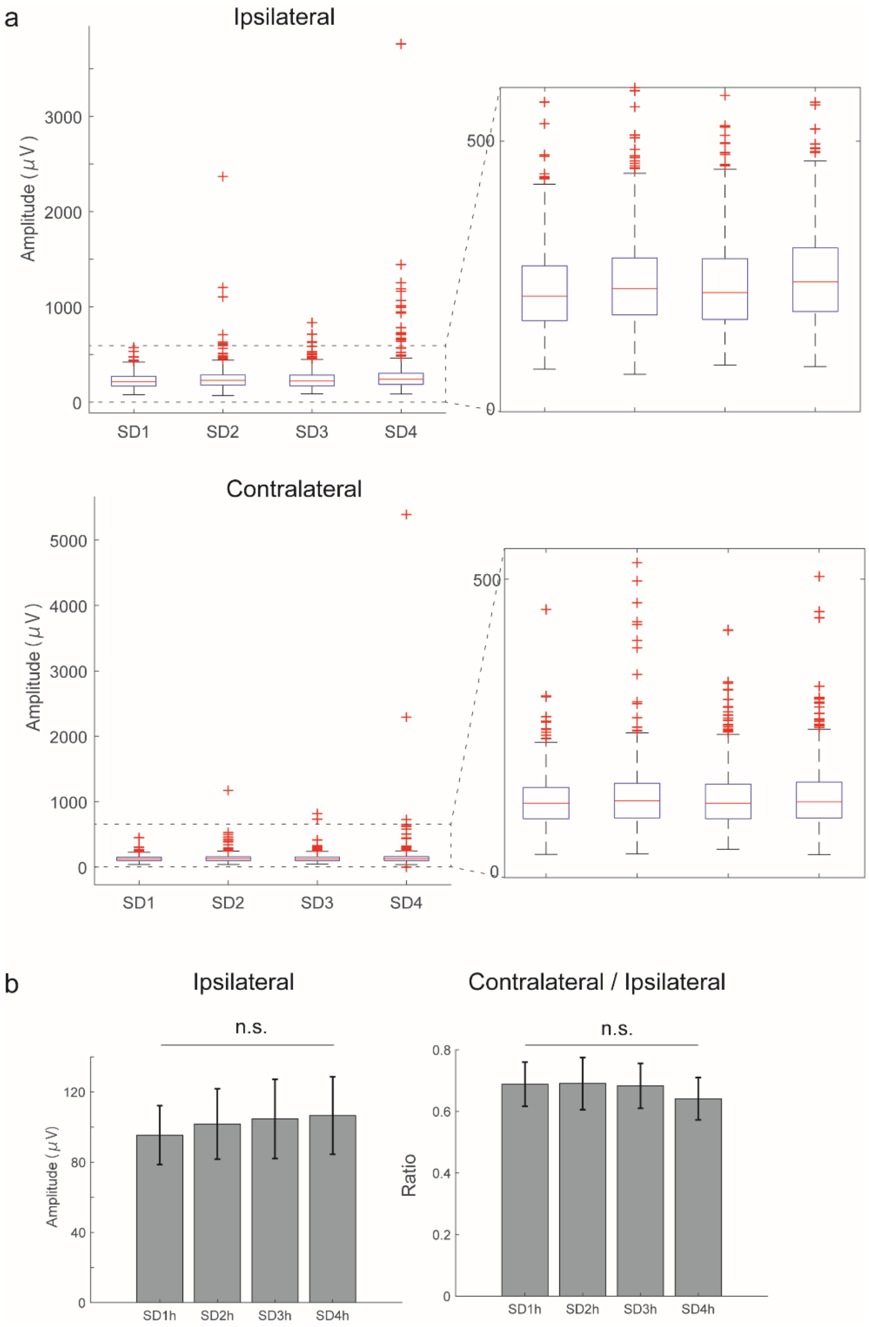

Figure S3.

a) Box plot of the response amplitude averaged in every 1 h during sleep deprivation of one animal (blue box: 25% and 75% quintile, red line: median, red plus: outliers). b) Average of the median LFP responses in waking during sleep deprivation error bars show standard deviation. (3 recordings, repeated measurement ANOVA).

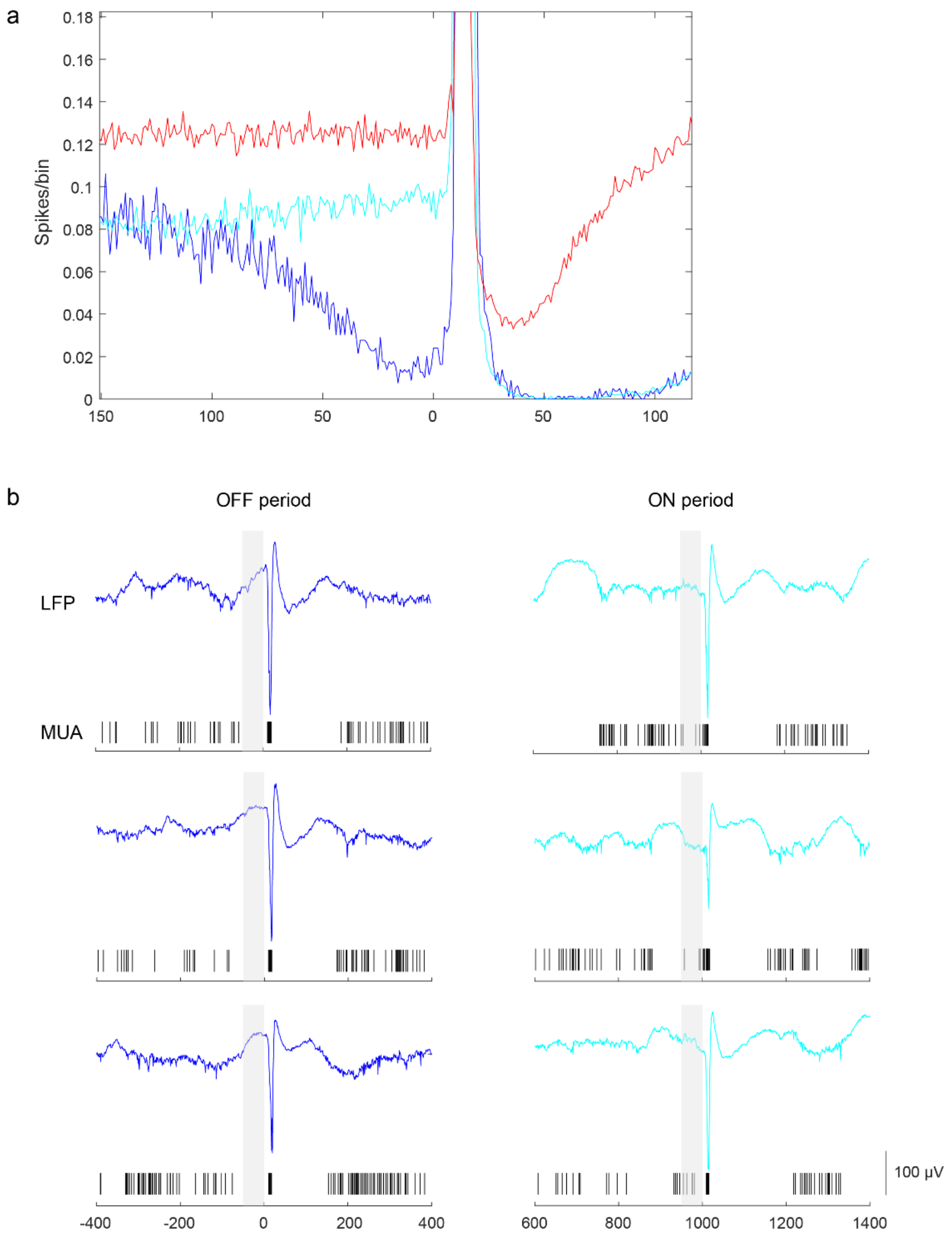

Figure S4.

a) High resolution of the MUA histogram (1 ms bin) with the light stimulation time point at 0 (red: wake, dark blue: OFF period, light blue: ON period). Note the low unit activity level in the OFF stimulation trace just before stimulation. b) 3 example traces which were categorized as OFF (left, dark blue) or ON (right, light blue) at the time of stimulation. Note the characteristic upward deflection
